# RE: Efficacy of botulinum toxin type A 100 units versus 200 units for treatment of refractory idiopathic overactive bladder

**DOI:** 10.1590/S1677-5538.IBJU.2015.0726

**Published:** 2016

**Authors:** Osama Abdelwahab, Hammouda Sherif, Tark Soliman, Ihab Elbarky, Aly Eshazly

**Affiliations:** 1 Urology department, Faculty of Medicine, Benha University, Egypt

*To the editor,*

We read with great interest in the article “Efficacy of botulinum toxin type A 100 Units versus 200 units for treatment of refractory idiopathic overactive bladder”. Osama Abdelwahab, et al. (1) nicely presented the treatment outcomes in this well conducted randomized controlled study.

Lack of voiding diary is one of the weak points of the article. Number of incontinence episode or functional bladder capacity is important in the evaluation of OAB. In addition, the mean pretreatment cystometric capacities in group A and B were 277.7±75.29 and 289.2±70.83ml, respectively. The high mean values mean that some of the patients actually had large cystometric bladder capacity before BoNT-A treatment and that they might not gain benefits from this intervention. So, it would be great to see the results in the subgroups of various functional or cystometric bladder capacity.

Meng-Jen Huang, MD Shag-Jen Chang, MD *and* Stephen Shei-Dei Yang, MD *Department of Surgery* *Taipei Tzu Chi General Hospital* *New Taipei, Taiwan No.289* *Jianguo Rd., Xindian Dist.* *New Taipei City 23142, Taiwan E-mail: krissygnet@yahoo.com.tw*
